# Assessing AI Awareness and Identifying Essential Competencies: Insights From Key Stakeholders in Integrating AI Into Medical Education

**DOI:** 10.2196/58355

**Published:** 2024-06-12

**Authors:** Julia-Astrid Moldt, Teresa Festl-Wietek, Wolfgang Fuhl, Susanne Zabel, Manfred Claassen, Samuel Wagner, Kay Nieselt, Anne Herrmann-Werner

**Affiliations:** 1 Tübingen Institute for Medical Education, University of Tübingen, Tübingen, Germany; 2 Institute for Bioinformatics and Medical Informatics, University of Tübingen, Tübingen, Germany; 3 Department of Computer Science, University of Tübingen, Tübingen, Germany; 4 Department of Internal Medicine, University Hospital of Tübingen, Tübingen, Germany; 5 Board of the Faculty of Medicine, University of Tübingen, Tübingen, Germany; 6 Department of Internal Medicine VI - Psychosomatic Medicine and Psychotherapy, University of Tübingen, Tübingen, Germany

**Keywords:** AI in medicine, artificial intelligence, medical education, medical students, qualitative approach, qualitative analysis, needs assessment

## Abstract

**Background:**

The increasing importance of artificial intelligence (AI) in health care has generated a growing need for health care professionals to possess a comprehensive understanding of AI technologies, requiring an adaptation in medical education.

**Objective:**

This paper explores stakeholder perceptions and expectations regarding AI in medicine and examines their potential impact on the medical curriculum. This study project aims to assess the AI experiences and awareness of different stakeholders and identify essential AI-related topics in medical education to define necessary competencies for students.

**Methods:**

The empirical data were collected as part of the TüKITZMed project between August 2022 and March 2023, using a semistructured qualitative interview. These interviews were administered to a diverse group of stakeholders to explore their experiences and perspectives of AI in medicine. A qualitative content analysis of the collected data was conducted using MAXQDA software.

**Results:**

Semistructured interviews were conducted with 38 participants (6 lecturers, 9 clinicians, 10 students, 6 AI experts, and 7 institutional stakeholders). The qualitative content analysis revealed 6 primary categories with a total of 24 subcategories to answer the research questions. The evaluation of the stakeholders’ statements revealed several commonalities and differences regarding their understanding of AI. Crucial identified AI themes based on the main categories were as follows: possible curriculum contents, skills, and competencies; programming skills; curriculum scope; and curriculum structure.

**Conclusions:**

The analysis emphasizes integrating AI into medical curricula to ensure students’ proficiency in clinical applications. Standardized AI comprehension is crucial for defining and teaching relevant content. Considering diverse perspectives in implementation is essential to comprehensively define AI in the medical context, addressing gaps and facilitating effective solutions for future AI use in medical studies. The results provide insights into potential curriculum content and structure, including aspects of AI in medicine.

## Introduction

### Background and Significance of AI in Medicine

In 1966, the architect Cedric Price [1] posed the provocative question, “Technology is the answer, but what was the question?” to encourage his lecture audience to explore, question, and reconsider the impact of technological progress. More than 50 years later, this question remains as relevant as ever. One might similarly ask today, “The answer is AI, but what was the question?” The health care sector is currently undergoing a significant transformation process characterized by the increased integration of digital technologies [2-4]. German clinics have been incorporating computer-driven clinical decision systems, such as the electronic patient record and other digital health tools, that can analyze data, identify patterns, and make decisions based on that data [3]. These intelligent systems can improve health care efficiency, accuracy, and quality while potentially reducing the burden on medical personnel [5,6]. Artificial intelligence (AI) technologies are already being implemented in various aspects of medical practice. For instance, they are used in imaging diagnostics where AI algorithms help analyze medical images [7]. Dictation systems with speech recognition powered by AI are also used, and AI chatbots are deployed to assist doctors and patients by providing appointments and information [8-10]. A range of sensor-based wearables, such as fitness trackers, smartwatches, and health apps, is already used in people’s daily lives. These devices use AI-supported algorithms to gather and analyze health data, including heart rate, sleep patterns, activity levels, and calorie consumption. Based on this information, personalized recommendations can be made to help individuals improve their well-being [11]. Although the use of medical AI systems remains in its early stages, ongoing research and development efforts are being undertaken worldwide. As technology rapidly advances, AI will increasingly play a crucial role in the future of health care [12,13]. This also requires restructuring medical curricula to adapt to dynamic technological advances to prepare students for the changing structures of medical practice [14,15].

Traditionally, medical education has focused on providing students with comprehensive knowledge of medical practices, diagnostic procedures, and treatment methods. Additionally, the effective use of AI in the medical field requires not only developing the necessary technological advances in AI applications but also ensuring that future physicians possess the required skills and expertise to effectively apply these technologies [16-18]. Therefore, it is crucial to consider integrating AI into the medical curriculum and determine how this technology can be effectively incorporated to benefit students and patients [19-21]. However, studies indicate that the integration of AI into the medical curriculum to enhance understanding of AI algorithms and optimize their use remains in its early stages, particularly in Germany [22-24]. Some institutions have developed specific courses and training programs to enhance medical students’ knowledge and skills in AI [25-27].

### Research Objectives and Research Questions

Given the complex and rapidly evolving nature of AI, no standardized definition or structured learning objectives currently exist regarding the specific AI topics medical students should be familiar with. Several studies emphasize the importance of understanding the fundamentals of AI and data science, mathematical concepts, and related ethical and social issues [26,28]. Medical students should also develop skills in interpreting AI models and be familiar with machine learning, deep learning, and data analytics to apply AI in clinical practice [29].

As part of a project, “TüKITZMed – Tübingen KI – Trainingszentrum für die Medizin” (Tübinger AI Training Center for Medicine), funded by the German Federal Ministry of Education and Research (16DHBKI086), a comprehensive needs assessment was conducted involving various stakeholders to understand the requirements and skills for integrating AI into the medical curriculum following step one of Kern’s 6-step approach [30]. The project “TüKITZMed” aims to develop and establish a cross-faculty interprofessional curriculum focused on “AI in medicine” providing students with a comprehensive understanding of the topic across different levels and disciplines. This curriculum serves as a pioneering example of integrating AI into academic programs, offering students opportunities for both theoretical learning and practical application, thereby facilitating the transfer of knowledge into real-world contexts. This study aimed to investigate essential AI knowledge for medical education curricula, identify necessary competencies through stakeholder input, and address potential gaps in learning opportunities. Involving different stakeholders offers diverse perspectives based on their roles and experiences. This approach helps identify relevant AI competencies and appropriate teaching formats, addressing unmet needs and challenges associated with implementing AI-focused learning opportunities in medical education [31].

Therefore, this paper aims to address the following research questions regarding assessing AI awareness and identifying essential competencies:

- How familiar are the different stakeholders with AI in general?
- Which specific aspects and topics related to AI are viewed as important?
- What competencies are crucial for medical health students?

## Methods

For a comprehensive understanding of AI and to address various aspects relevant to the surveyed stakeholders’ perspectives on an AI curriculum, an exploratory research approach using semistructured interviews was chosen. The incorporation of narrative-generating guideline-supported questions aimed to establish a structured framework for investigating research interests while also allowing flexibility to uncover new and insightful content [32].

### Study Design and Setting

This qualitative study approach followed the Standards for Reporting Qualitative Research [33]. It was performed at the Medical Faculty and the Faculty of Science of the University of Tübingen as part of the TüKITZMed project.

### Sample Selection and Recruitment

Semistructured interviews with 38 stakeholders involved in the implementation process of AI in medical curricula were conducted to gather diverse perspectives and insights. Relevant stakeholders were characterized as individuals impacted by the integration of AI in health care, those with professional experience with AI technology, and those who had previously encountered AI applications in the medical sector. The stakeholder groups comprised the following: 6 lecturers, 9 clinicians, 10 students, 6 AI experts, and 7 institutional stakeholders. The interview guide followed the guiding research questions for the needs assessment [34]. An illustrative interview guide is provided in Multimedia Appendix 1.

The selection of stakeholder groups was based on their crucial role in the field of medical education and their diverse perspectives. For participant recruitment, we used an open approach, reaching out to stakeholders primarily via email after identifying relevant stakeholder groups for our research inquiries. Inclusion criteria included individuals working with AI in the medical context or possessing relevant expertise, especially clinicians and AI experts. Due to the project’s regional focus, only stakeholders from the local area were approached. Recommendations, referrals, and requests within working groups or via email forwarding were also used. Potential participants were also approached at conferences.

#### Lecturers

Educators’ perspectives are required, as integrating AI into medical education is an unprecedented challenge with no clear guidelines. Even if consensus is reached on exactly what should be taught to medical students, it remains daunting to determine how best to teach it. The experience of educators—especially those familiar with medical students—is therefore imperative in the process [35,36].

#### Students

Health care students’ perspectives (eg, on human medicine, medical technology, and molecular medicine) are central to integrating AI into medical education since the curricula should ultimately be designed to serve their educational needs. Therefore, assessing their current state of knowledge, attitudes, and heterogeneity across different student populations is an important step in adequately addressing the educational needs for medical AI and integrating it such that students will benefit from it [18,37].

#### AI Experts

AI experts have long-standing knowledge and expertise in the field. Engaging with them provides valuable insights into the latest developments, trends, and best practices in AI. These experts offer a thorough understanding of AI concepts, applications, and their potential impact on health care [36,38].

#### Clinicians

Involving medical staff in developing medical AI helps find clinical value while protecting patient safety. Moreover, medical staff know the data well and are thus the only ones who can detect the bias or impracticality of AI. Additionally, medical experts play a key role in teaching real-world medical applications of AI, as they have the experience and skills. Thus, their perspectives are relevant to the integration of AI into education and practice since they can inspire other medical workers to engage with it [39].

#### Institutional Stakeholders

The perspectives of institutional stakeholders are crucial for driving change in medical education. These individuals hold key positions within educational or health care institutions and are actively involved in implementing AI within the medical curriculum. Such stakeholders, including deans, program coordinators, and administrative staff, possess specific training and qualifications relevant to their roles, playing an essential part in shaping educational strategies and health policies. Given the already full capacity of medical curricula, their support and expertise are necessary for a meaningful integration of AI. Additionally, institutional stakeholders provide an important framework for continuously monitoring and reevaluating the implementation of AI in medical curricula to ensure its utility and quality [18,40,41].

### Data Collection

Semistructured guided interviews were chosen as they allow a flexible participation-centered approach and in-depth exploration of the topic, capturing the diverse perspectives of the stakeholders involved [42]. The semistructured guided interviews were conducted from August 2022 to March 2023, either face-to-face or via videoconference. All interviews were audio recorded and transcribed verbatim for analysis. Before participation, written informed consent was obtained from all the interviewees. The resulting code system for analysis was consolidated and summarized.

### Data Analysis

The transcripts were analyzed according to the principles of content structuring analysis, as outlined by Kuckartz [43]. After the interviews were transcribed, independent researchers thoroughly reviewed them. The category system for the analysis was developed using the semistructured guiding questionnaire as a basis (inductive approach) and systematically coded using the MAXQDA 2022 software program (VERBI GmbH). As the coding process progressed, new categories emerged to include additional aspects and themes discussed in the interviews. This step enabled flexibility and openness to new insights that transcended the initially defined structure (deductive approach) [44]. Collectively, we presented outcomes derived from diverse stakeholders. We systematically addressed varying perspectives within or across these cohorts, emphasizing their respective relevance. Our presentation includes literal quotations, preserving the original expressions translated from German to English.

### Ethical Considerations

The study received ethical approval from the Ethics Committee of Tübingen Medical Faculty (467/2022BO2). Participation was voluntary. All participants were informed of the purpose of the study and provided informed consent before data collection. The confidentiality of all data was ensured, and all responses and data were kept anonymous. The participants had the right to withdraw from the study at any time. Participants did not receive any compensation.

## Results

### Overview

The ages of participants ranged from 19 to 59 (mean 38.5, SD 9.7, SEM 1.6) years, with data provided by 36 individuals. Regarding sex distribution, there were 26 male and 12 female participants. Through a structuring content analysis, we systematically derived 6 primary categories with a total of 24 subcategories from the entire data set.

### Presentation of Stakeholder Perspectives and Expectations of AI

The analysis of the stakeholders’ statements revealed several commonalities and differences regarding their understanding of AI.

#### AI as a Tool

In terms of commonalities, the actors viewed AI primarily as a tool that can analyze and process large amounts of data:

*For me, it’s mainly a toolbox, a toolkit. These are technologies that help us.*

[RR22T, expert]

*An AI can process much more data at once than a human could.*

[KC10S, student]

*A way to predict things, that is, to predict data based on existing data and also to apply techniques that support us to categorize, assess, simulate, and also predict things in terms of the future.*

[KU512S, institutional stakeholder]

#### AI as a Medical Assistant

Additionally, stakeholders emphasized the potential benefits of using AI to assist in medicine, whether in supporting diagnostic and treatment decisions or more efficiently mining clinical data:

*In the context of medicine, probably so therapy decisions, more efficient evaluation of clinical data.*

[AB001, lecturer]

*To this, I can think of automation and standardization of processes but also help in an increasingly complex clinical situation with many parameters and many possibilities relevant for decision-making by doctors involved in therapy and diagnosis.*

[RW01R, institutional stakeholder]

#### The Technical Understanding of AI

A focus on understanding AI is also related to the technical background of AI technology and the roles of mathematical-statistical models, data, and algorithms:

*AI is learning systems that fit a model with computations to data.*

[PG49B, expert]

*AI is about programs and algorithms that improve with more data and data processing*.

[CCHU7, clinician]

#### Differences in the Understanding of the Term and Difficulties in Formulating a Definition

However, differences in the understanding of AI between the stakeholders interviewed also became apparent. These disparities manifested in three key areas: challenges in formulating a concise definition and description of AI, diverse perspectives and expectations regarding AI capabilities, and varying emphasis on the medical fields where AI is anticipated to make significant progress.

*The name “AI” is misleading because there is currently no computer application that is actually intelligent. Rather, it refers to algorithms with high computing power that enable computers to process larger amounts of data than before and possibly even evolve themselves.*

[RH01W, lecturer]

*Artificial intelligence is a difficult term. It suggests that human intelligence is extended and artificially subsumed.*

[EJ12B, institutional stakeholder]

*Understanding AI is complex and broad. There is AI that learns itself and AI that still needs to be monitored. AI is otherwise a kind of automated analysis.*

[HT02B, clinican]

#### Varying Perspectives and Expectations on the Potential and Capabilities of AI

While some may view AI capabilities more optimistically or comprehensively, others emphasize the limits and specialized functions AI can possess. For example, the students tended to view AI as efficient data processing, while the experts emphasized AI’s capacity for revealing hidden patterns and simulating complex scenarios. The students emphasized that AI lacks a creative process and cannot engage in creative thinking. They focused on AI’s ability to efficiently process and analyze large amounts of data. The experts discussed AI’s ability to identify scientific connections, structures, and patterns that may be imperceptible to humans. They recognized AI’s potential to simulate complex scenarios and discover novel insights from data.

*Systems that can recognize scientific relationships, structures, and patterns that are not discernible to humans.*

[PG49B, AI expert]

*AI is not intelligent because no creative process can take place in it. It can quickly and efficiently draw connections from large amounts of data.*

[YG30B, student]

*In most cases, however, AI is about better evaluating large amounts of data and modifying it through self-learning algorithms. Human intelligence can understand and solve problems through creativity and think outside of rules – so it works differently. However, algorithms can do other things better than humans.*

[EJ12B, institutional stakeholder]

*AI will determine everyday life, but also medicine more and more. Nowadays, one is often confronted with the topic, and one should deal with it*.

[IE13H, clinician]

#### Different Emphases of the Potential Areas of Application

Although some alignment existed in the perceptions of the potential uses of AI, each group had its own focus on specific applications of AI in health care, depending on profession and discipline. AI experts and lecturers emphasized the significance of the technical dimensions of AI, including the essential roles of algorithms, data processing, and AI models in medical research and practice. Students underscored AI’s role in aiding health care professionals, while clinicians concentrated on the clinical sphere of AI, particularly its contributions to diagnostics, treatment decisions, and data processing. Additionally, institutional stakeholders highlighted the potential for increased efficiency in health care by implementing AI solutions. They engaged in discussions concerning the pragmatic integration of AI to strengthen clinical decision-making processes and optimize operational workflows.

*But to provide a definition..., so what we’re actually doing is, we’re learning relationships between input, certain inputs, and certain outputs: What is the relationship between an X-ray image and a diagnosis? And this correlation, you can then learn it using, for instance, a neural network, and then apply it to unseen X-ray images.*

[MF08Z, lecturer]

*To simplify processes, so to speak, to facilitate and automate simple processes that would normally take a lot of time for us humans. The machine can recognize complex relationships that we as humans either cannot comprehend or, as mentioned, would take a long time to understand. For example, in my case, it’s radiation therapy in radio oncology, where there are many processes that take a long time or are, as I said, very complex because, in medicine, we naturally have many intricate aspects and influences on the patient that we need to consider. And a machine can handle this quite well, as it can analyse and evaluate these various data effectively, essentially.*

[EK05B, student]

*An evolving field, which is already partially present in clinical reality. This involves automation and standardization of processes, as well as assistance in an increasingly complex clinical environment with numerous parameters and numerous possibilities that are relevant for decision-making by physicians and individuals involved in therapy and diagnosis.*

[RW01R, institutional stakeholder]

### Identification of AI Competencies and Implications for Medical Curriculum

We identified 4 main categories of implementation needs (Textbox 1)

Textbox 1. **Identified main categories of implementation needs: conceptual designs for an artificial intelligence curriculum.**

**Possible curriculum contents, skills, and competencies**

- Basic understanding and sense of technology
- Data literacy
- Morality and ethics
- Opportunities and risks
- Digital literacy
- Application of software
- Data privacy
- Understanding of medical test results

**Programming skills**

- Voluntary: Having programming skills is optional. Although they are not mandatory, having them is beneficial.
- Not required: Programming skills are unnecessary. However, if one possesses such skills, that is acceptable.
- Required: Programming skills are mandatory. Basic or advanced programming proficiency is expected for participation.

**Curriculum scope**

- Adapted to the time available
- Intensive engagement

**Curriculum structure**

- Lecture
- Seminar
- Interactive exercises
- Consolidation for specialization
- Basics as lecture with exercise
- No opinion due to lack of experience
- Interdisciplinary
- Adapt curriculum dynamically according to relevance

#### Possible Curriculum Contents, Skills, and Competencies

This main category covers a range of topics, including possible curriculum components, skills, and abilities students should learn regarding AI in medicine.

##### Basic Understanding and Sense of Technology

The first subcategory addresses the need for medical students to develop a basic understanding of the fundamental principles and concepts of AI. This includes understanding the essential mechanisms of machine learning algorithms and acquiring basic knowledge of mathematical computer science.

And I believe that what would be important to develop a bit of an understanding of how the technology actually works....So, I don’t think that you can teach all of that to medical students from the ground up in theoretically well-founded way with linear algebra and so on. But I do think that it’s quite impossible to offer an applied course where they can practise and play around with it, get a sense of how technology functions.

[MF08Z, lecturer]

##### Data Literacy

The data literacy category describes the need to provide medical students with the skills and knowledge required to effectively handle and interpret data in the context of AI applications in the field of medicine.

Data quality is crucial. In my view, all the methods of machine learning are secondary....But the most important thing is truly obtaining high-quality data, understanding how to work with data, understanding implications of the data.

[DD21S, expert]

##### Morality and Ethics

The morality and ethics subcategory is dedicated to providing students with an in-depth understanding of the ethical considerations associated with integrating AI into the medical field. It aims to develop a keen awareness of the ethical responsibilities associated with AI advances in the medical field.

Ethics comes to mind...I consider it a highly relevant aspect because AI tools that are intended for future use in medicine are, in my opinion, closely tied to patients, to human beings; potentially, these tools could make life and death decisions, and in that regard, I would argue that entirely different requirements for quality assurance, ethical standards and checks and boundaries need to be in place for these tools....That should be covered during education*.*

[RW01R, institutional stakeholder]

##### Opportunities and Risks

The opportunities and risks subcategory includes aspects related to awareness of the potential benefits and challenges associated with integrating AI into health care. Medical students should be empowered to navigate the complex landscape of AI in medicine by not only recognizing the potential benefits but also being able to address challenges, make informed decisions, and maintain vigilance concerning its capabilities and limitations.

Also understanding how to interact with AI. To what extent can I trust the AI, the outcomes it produces? How can I collaborate effectively with it? What do I need to operate a good AI, and where can it also be deployed?

[SA01R, student]

##### Digital Literacy

Several actors addressed the need for medical students to be equipped with the skills required to navigate and use digital technologies effectively. This includes developing proficiency in using AI-powered diagnostic support tools, including the ability to interpret and apply AI-generated diagnostic insights. This also extends to understanding and implementing adaptive learning methodologies and leveraging telemedicine for remote patient care.

What is also important to me, when we talk about the topic of artificial intelligence, is that we first discuss the fundamental aspects of digitization and the necessary measures for healthcare, research, and education....We are delving into a very specific topic, but we still lack some of the foundational knowledge.

[EH07S, institutional stakeholder]

##### Application of Software

The application of software subcategory concerns equipping individuals with the ability to effectively use software tools, particularly in the context of AI development and implementation.

Medical students often lack knowledge in this area. Therefore, I believe it’s important for them to have hands-on experience of training a neural network themselves.

[MF08Z, expert]

##### Data Privacy

The data privacy category describes the aspects the actors mentioned to give students the expertise to address the ethical and legal issues related to data privacy in AI applications. By mastering data management practices and understanding the legal framework, students ensure patient data is managed safely, impartially, and ethically in the context of AI integration.

The topic of data privacy should definitely be included in the curriculum because the “who” question of how to do this, how it’s trained and on which data sources, most of it needs to be anonymized.... Ethics and data privacy are two significant components that need to be integrated, unfortunately or fortunately*.*

[HT02B, clinician]

##### Understanding of Medical Test Results

The last subcategory summarizes the need for in-depth expertise in AI-driven application outcomes, particularly in the context of medical tests. Students must develop a profound understanding of the insights and outcomes produced by AI applications, including acquiring the expertise to thoroughly analyze and interpret results derived from AI-powered processes.

The most important aspect of AI is understanding the basis on which decisions are made*.*

[JJ22D, clinician]

### Programming Skills

Clinicians stated that a programming course for medical students should not be mandatory due to overload but could be offered as an elective. Instead, AI experts should be involved due to their expertise in AI applications in the medical field. However, a basic understanding of programming should be acquired early, especially for those who are interested and want to pursue a science career. Most clinicians surveyed opposed including programming skills in the curriculum.

Some lecturers also disagreed with integrating programming knowledge into the curriculum due to student overload. It was emphasized that it is unnecessary for physicians to be able to program neural networks, for example, but that a basic understanding of application knowledge should be established. However, some also emphasized that programming skills and basic computer science knowledge are important, including Python, R, and a theoretical understanding of algorithms. Opinions on the topic were divided and varied depending on the respondents’ areas of expertise.

The students interviewed also believed programming skills should be offered to those interested but should not be mandatory. The majority rejected the integration of programming skills into the curriculum, as they are considered too extensive for medical studies and appear to be of minimal relevance to practical application.

AI experts emphasized that physicians need a basic understanding of AI to build confidence in AI applications. Opinions on programming skills were divided, with some considering simple programming skills helpful. Institutional stakeholders also believed medical students do not necessarily need to know how to program but should have field competence in programming. It is expected that not all medical students will be able to program or develop learning methods themselves. However, a basic understanding of programming is viewed as increasingly essential.

### Curriculum Scope

The design and scope of AI courses in medicine vary. Including a small section in the curriculum, adapted to the students’ abilities, is recommended. For radiologists and image-based diagnosticians, intensive exposure to AI is useful. This should cover a practical application with real medical data to show the application’s relevance. Online courses for practicing physicians were suggested to learn the basics.

### Curriculum Structure

Regarding AI education in medicine, two main approaches are being considered: lectures and seminars. For lectures, the focus is on introducing mandatory courses blending theory with practical applications. Seminars are viewed as a means to give students early practical experience, enhancing their engagement. Due to the subject’s complexity, lecturers are advised to emphasize fundamentals and incorporate concrete examples. However, it is noted that students might find lectures overwhelming, especially without mandatory exams or regular attendance.

Stakeholders emphasized the need for a practical and interactive design when conveying AI content, with clear applications that allow students to experiment for maximum learning impact. Basic AI competencies should be part of the standard medical curriculum, with options for specialization for those interested, particularly those pursuing a scientific career.

Incorporating AI competencies into medical education is recommended, either through a holistic course or integration into subject-specific areas. Interdisciplinary, research-oriented, and application-oriented seminars and workshops should be established to provide in-depth knowledge. In the future, the curriculum will require substantial restructuring to effectively integrate evolving AI content. Given the rapidly changing nature of AI, the curriculum must remain adaptable.

**Figure 1.**
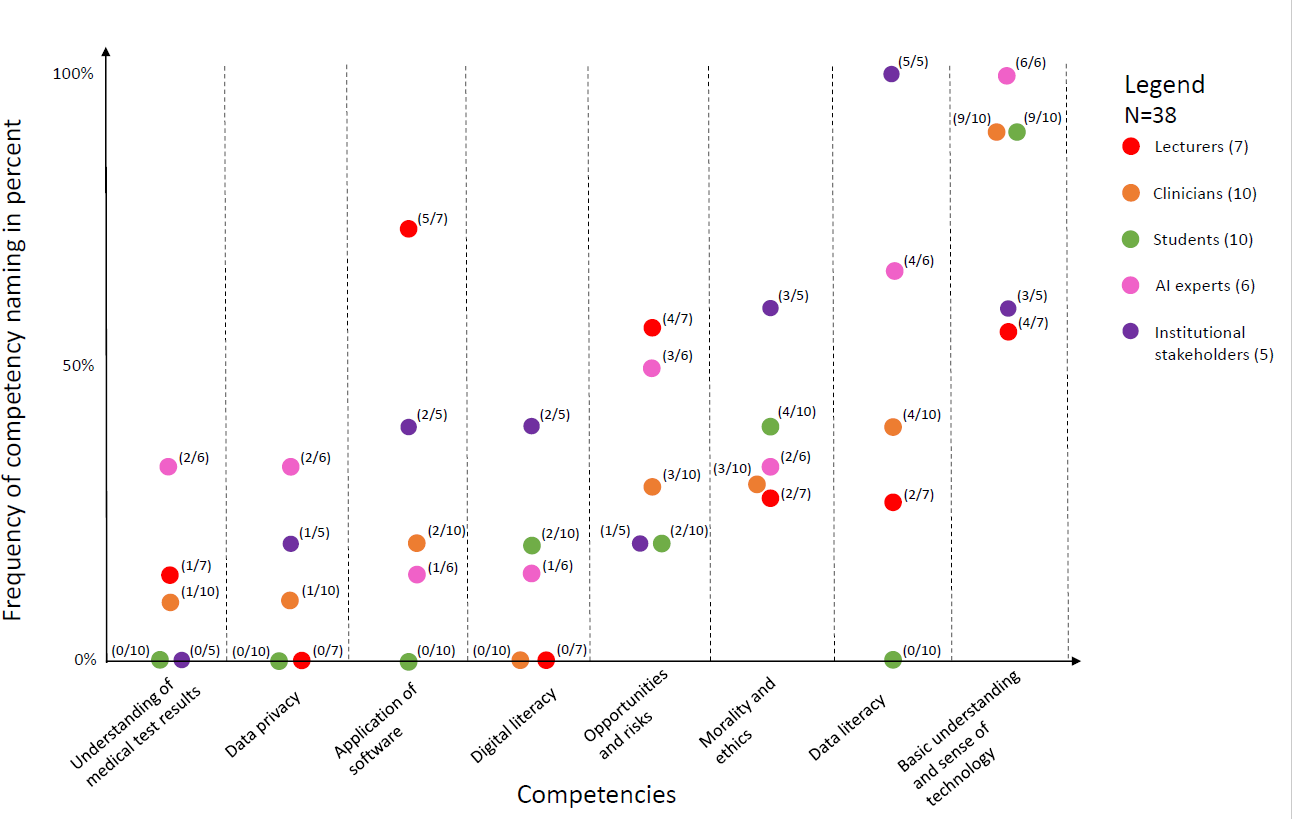
Main categories by occurrence of mentions per stakeholder group (qualitative content analysis). The presentation of the primary categories in the qualitative content analysis is based on the frequency of mentions per stakeholder group rather than a quantitative analysis of frequency distribution. AI: artificial intelligence.

As shown in Figure 1, the competencies highlighted by different stakeholder groups reveal a range of perspectives and priorities. These focus on the frequency of topics falling into these main categories, offering a nuanced understanding of the thematic landscape.

For example, each stakeholder group highlights the significance of possessing a basic understanding of AI and an awareness of AI-supported applications. Similarly emphasized is the importance of gaining a principal perspective on the opportunities and limitations of AI in medicine, as well as addressing ethical considerations and potential dilemmas. AI experts also emphasized topics such as data literacy, fundamental computer science and mathematics skills, and gaining an overview of potential application areas, while institutional stakeholders focused on interdisciplinary approaches and legal requirements.

## Discussion

### Principal Findings

The insights gained from the study of stakeholder statements provide valuable perspectives on the different views and interpretations of AI. This provides the basis for answering two central research areas. The first is the understanding of AI, particularly how different interest groups perceive this technology. Second, the focus is on the AI skills that should be taught in medical studies. The different stakeholder groups, including lecturers, health care students, AI experts, institutional stakeholders, and clinicians, contributed to a multifaceted picture. The analysis highlighted similarities and differences in the perception of AI by the various stakeholder groups. These findings from our investigation correspond to step one of Kern’s 6-step approach. They are crucial for discussions on implementing AI in health care and underline the need for clear communication, education, and a common understanding of terminology.

### Key Competencies for Health Science Students and the Need for a Common Understanding of AI

The qualitative content analysis revealed a broad spectrum of perceptions of AI among the interviewees. Especially in rapidly advancing fields such as AI, creating and maintaining a common language is essential to enable effective collaboration between different stakeholders. AI is a broad field incorporating many technologies and methods. When introducing AI into the health care system, it is important to acknowledge that different stakeholders involved may have different perceptions of the term AI. A clear definition of this complex term helps prevent misunderstandings, as the field of AI is expansive, encompassing various technologies and methodologies [45,46].

Depending on the contextual background and prior knowledge of the individuals, different descriptions and emphases emerged in the definition of AI. Similarly, ideas concerning the opportunities and limitations of AI in medicine varied depending on individual backgrounds. If consensus is lacking on what AI means in the context of medical education, this can lead to confusion and disagreement on which AI competencies are essential for medical students. This lack of clarity can hinder the development of standardized curricula and educational programs related to AI in medical education, especially when different stakeholders with different backgrounds might be mixing the AI terminologies “strong AI” and “weak AI” [47,48]. Therefore, a clear understanding of the implications and limitations of AI in the medical field is crucial for establishing effective educational guidelines.

Considering these diverse perceptions of AI are particularly relevant when teaching AI skills to medical students. The diversity in the understanding of AI only emphasizes the complexity of the topic; thus, a strong interdisciplinary approach is necessary. Collaboration between physicians, computer scientists, ethicists, and other experts is essential to fully understand the challenges and opportunities of AI in the medical context. For instance, this becomes particularly important when determining which AI applications can enhance the learning experience in specific medical specialties [41].

The diverse perspectives among stakeholders indicate a consensus regarding the essential competencies for health care students concerning AI integration. Recurring themes include practical experience, fundamental digitization knowledge, ethical considerations, and a profound understanding of data and technology. Balancing these competencies is critical to preparing future health care professionals to effectively use AI while maintaining ethical standards and a patient-centered approach. Continued collaboration between stakeholders and the adaptability of medical education curricula will play a key role in achieving these goals.

As illustrated in Figure 1, the stakeholders exhibit substantial diversity in their prioritization of topics and skills, highlighting significant variations in the perceived importance of AI integration into the curriculum. The discussion underscores the importance of a comprehensive approach to AI education in medicine, incorporating practical experience, ethical considerations, and a nuanced understanding of AI’s role in health care. In the context of AI competencies for medical students, they must possess not only medical knowledge but also basic knowledge of AI applications and data literacy, as AI in medicine is becoming increasingly data intensive. The ability to accurately evaluate, manage, and safeguard medical data is essential to ensure that AI technologies can be effectively and ethically deployed in patient care. Therefore, collaboration between stakeholders is essential to develop a curriculum equipping future medical professionals with the necessary competencies to navigate the complexities and opportunities presented by AI in medicine.

### The Impact of AI on Shaping Individual Behavior and Societal Outcomes in Medical Training

Since the 1980s, it has been recognized that the introduction of new technologies such as AI does not occur in isolation or independent of societal influences, contrary to the earlier assumption of technological determinism [49,50]. Technology development is shaped by social construction and negotiation processes, where technology emerges as a social construct through human action and influences societal structures and institutions [51]. Interactions related to the introduction of AI in health care can significantly impact how patients are treated and how medical information is used [52]. This concerns not only introducing a new technology per se but also ensuring that it has long-term and positive effects. A key aspect is ensuring that the implementation of AI in health care respects and considers the existing values, norms, and needs of society. Therefore, ethical compatibility and adherence to societal standards are fundamental [53].

Furthermore, AI technologies influence not only medical knowledge but also how doctors, patients, and other stakeholders in health care understand and define their roles. Comprehensive integration of AI requires a holistic approach that not only relies on technological advances but also appropriately considers social dynamics and human aspects.

Our analysis also illustrates the broad understanding of AI, a disparate overall picture of the necessary AI competencies for future medical professionals, and the possibilities and risks associated with implementation. While it is a hot topic among AI experts, health care students are not yet fully aware of the significance of AI, although the technology is expected to enter their professional lives in the future [31,54]. In medical education, students should actively engage with AI, moving beyond passive roles. As well as regulatory, technical, and ethical aspects, it is crucial to consider the sociotechnical dimensions of AI. This is vital, as students must cultivate not only a deep understanding of AI but also an awareness of its societal complexities. For example, Sartori and Bocca [55] emphasize that narratives, whether from the media, scientific community, fiction, or other sources, significantly influence how society perceives and understands technology, including AI. These narratives contribute to the formation of shared understandings, values, and expectations about technology and its potential impact on society [55].

### Conclusion

The diverse perspectives on AI among the interviewees underline the requirement for a common language in this rapidly advancing field. Introducing AI into health care necessitates an awareness of varying stakeholder perceptions, emphasizing the importance of a clear definition to prevent misunderstandings. Individual backgrounds shape distinct descriptions and emphases in defining AI, leading to diverse ideas about its opportunities and limitations, particularly in the context of medical education. When teaching AI skills to medical students, it is essential to address this diversity and adopt a robust interdisciplinary approach to ensure future health care professionals acquire essential knowledge and skills. The results underscore the significance of a comprehensive AI education in medicine, integrating practical experiences, ethical considerations, and a nuanced understanding of AI’s role in health care. These competencies will enable medical students to critically evaluate AI technologies and use them responsibly in clinical practice, promoting a more informed and ethically sound integration of AI into health care. The lack of standardization in defining and teaching AI in medical education can lead to uncertainty and potential rejection of the technology. Closing this gap requires gaining insights into the knowledge and skills medical students should acquire regarding the use of AI in medicine. Future studies must focus on awareness of AI and perceived opportunities and risks associated with its implementation. This is also crucial for developing a holistic perspective on competencies within the medical curriculum.

### Limitations

While the qualitative nature of our study enabled in-depth exploration and rich insights into the stakeholder perceptions, the limitations associated with the sample size of 38 participants must be acknowledged. The findings may be context specific, and caution is warranted in generalizing beyond our studied group. Notably, some interviewees held dual roles, such as being both lecturers and clinicians. Due to practical constraints, they were interviewed in only one capacity, either as lecturers or clinicians. This limitation underscores the complexity of their perspectives, as their roles encompass multifaceted responsibilities. Using a partially standardized guiding questionnaire, participants were prompted to consider specific questions they might not have spontaneously discussed. While this may have influenced the direction of the conversation, we believe it encouraged participants to reflect. However, it must be acknowledged that a more comprehensive and representative understanding would require further exploration through a quantitative survey. Of note, a subsequent paper will address the opportunities and challenges associated with implementing AI in health care identified by the participating stakeholders.

## Supplementary material

10.2196/58355 Multimedia Appendix 1 Guiding questions for artificial intelligence experts (example).

## Abbreviations

AI: artificial intelligence

## References

[1] Price C (1979). Technology is the answer, but what was the question?. Pidgeon Digital.

[2] Stachwitz P, Debatin JF (2023). Digitalization in healthcare: today and in the future [Article in German]. Bundesgesundheitsblatt Gesundheitsforschung Gesundheitsschutz.

[3] Lohmann A, Schömig A, Beck S, Kusche C, Valerius B (2020). Digitalisierung, Automatisierung, KI Und Recht [Book in German].

[4] Lin B, Wu S (2022). Digital transformation in personalized medicine with artificial intelligence and the internet of medical things. OMICS.

[5] Briganti G, Le Moine O (2020). Artificial intelligence in medicine: today and tomorrow. Front Med (Lausanne).

[6] Fahy N, Williams GA, Habicht T (2021). Use of Digital Health Tools in Europe: Before, During and After COVID-19.

[7] Saw SN, Ng KH (2022). Current challenges of implementing artificial intelligence in medical imaging. Phys Med.

[8] Nadarzynski T, Miles O, Cowie A, Ridge D (2019). Acceptability of artificial intelligence (AI)-led chatbot services in healthcare: a mixed-methods study. Digit Health.

[9] Juluru K, Shih HH, Keshava Murthy KN (2021). Integrating AI algorithms into the clinical workflow. Radiol Artif Intell.

[10] Hornegger J, Knappertsbusch I, Gondlach K (2021). Arbeitswelt und KI 2030: Herausforderungen und Strategien für die Arbeit von morgen [Book in German].

[11] Mirmomeni M, Fazio T, von Cavallar S, Harrer S, Sazonov E (2021). Wearable Sensors: Fundamentals, Implementation and Applications.

[12] Rajpurkar P, Chen E, Banerjee O, Topol EJ (2022). AI in health and medicine. Nat Med.

[13] Ahuja AS (2019). The impact of artificial intelligence in medicine on the future role of the physician. PeerJ.

[14] Ng KH, Wong JHD (2022). A clarion call to introduce artificial intelligence (AI) in postgraduate medical physics curriculum. Phys Eng Sci Med.

[15] Mosch L, Agha-Mir-Salim L, Sarica MM, Balzer F, Poncette AS (2022). Artificial intelligence in undergraduate medical education. Stud Health Technol Inform.

[16] Han ER, Yeo S, Kim MJ, Lee YH, Park KH, Roh H (2019). Medical education trends for future physicians in the era of advanced technology and artificial intelligence: an integrative review. BMC Med Educ.

[17] Wallis C (2019). How artificial intelligence will change medicine. Nature.

[18] Wartman SA, Combs CD (2019). Reimagining medical education in the age of AI. AMA J Ethics.

[19] Dumić-Čule I, Orešković T, Brkljačić B, Kujundžić Tiljak M, Orešković S (2020). The importance of introducing artificial intelligence to the medical curriculum - assessing practitioners' perspectives. Croat Med J.

[20] Kundu S (2021). How will artificial intelligence change medical training?. Commun Med (Lond).

[21] McCoy LG, Nagaraj S, Morgado F, Harish V, Das S, Celi LA (2020). What do medical students actually need to know about artificial intelligence?. NPJ Digit Med.

[22] Grunhut J, Wyatt AT, Marques O (2021). Educating future physicians in artificial intelligence (AI): an integrative review and proposed changes. J Med Educ Curric Dev.

[23] Moldt JA, Festl-Wietek T, Madany Mamlouk A, Nieselt K, Fuhl W, Herrmann-Werner A (2023). Chatbots for future docs: exploring medical students' attitudes and knowledge towards artificial intelligence and medical chatbots. Med Educ Online.

[24] Lewis SJ, Gandomkar Z, Brennan PC (2019). Artificial intelligence in medical imaging practice: looking to the future. J Med Radiat Sci.

[25] Flasdick J, Mah DK, Bernd M, Rampelt F (2023). Micro-credentials and micro-degrees current developments and potentials for educational practice based on the example of the AI campus. ResearchGate.

[26] Hu R, Fan KY, Pandey P (2022). Insights from teaching artificial intelligence to medical students in Canada. Commun Med (Lond).

[27] Krive J, Isola M, Chang L, Patel T, Anderson M, Sreedhar R (2023). Grounded in reality: artificial intelligence in medical education. JAMIA Open.

[28] Karaca O, Çalışkan SA, Demir K (2021). Medical artificial intelligence readiness scale for medical students (MAIRS-MS) - development, validity and reliability study. BMC Med Educ.

[29] Sapci AH, Sapci HA (2020). Artificial intelligence education and tools for medical and health informatics students: systematic review. JMIR Med Educ.

[30] Thomas PA, Kern DE, Hughes MT, Tackett SA, Chen BY (2022). Curriculum Development for Medical Education: A Six-Step Approach.

[31] Nyein KP, Gregory ME (2023). Needs Assessment and Stakeholders in Medical Simulation Curriculum Development.

[32] Magaldi D, Berler M, Zeigler-Hill V, Shackelford TK (2018). Encyclopedia of Personality and Individual Differences.

[33] O’Brien BC, Harris IB, Beckman TJ, Reed DA, Cook DA (2014). Standards for reporting qualitative research: a synthesis of recommendations. Acad Med.

[34] Kallio H, Pietilä AM, Johnson M, Kangasniemi M (2016). Systematic methodological review: developing a framework for a qualitative semi-structured interview guide. J Adv Nurs.

[35] Abd-Alrazaq A, AlSaad R, Alhuwail D (2023). Large language models in medical education: opportunities, challenges, and future directions. JMIR Med Educ.

[36] Grunhut J, Marques O, Wyatt ATM (2022). Needs, challenges, and applications of artificial intelligence in medical education curriculum. JMIR Med Educ.

[37] Blease C, Kharko A, Bernstein M (2022). Machine learning in medical education: a survey of the experiences and opinions of medical students in Ireland. BMJ Health Care Inform.

[38] Katznelson G, Gerke S (2021). The need for health AI ethics in medical school education. Adv Health Sci Educ Theory Pract.

[39] Ganapathi S, Duggal S (2023). Exploring the experiences and views of doctors working with artificial intelligence in English healthcare; a qualitative study. PLoS One.

[40] Wartman SA, Combs CD (2018). Medical education must move from the information age to the age of artificial intelligence. Acad Med.

[41] Busch F, Adams LC, Bressem KK (2023). Biomedical ethical aspects towards the implementation of artificial intelligence in medical education. Med Sci Educ.

[42] Helfferich C, Baur N, Blasius J (2019). Handbuch Methoden Der Empirischen Sozialforschung [Book in German].

[43] Kuckartz U (2012). Qualitative Inhaltsanalyse: Methoden, Praxis, Computerunterstützung [Book in German].

[44] Mayring P (2019). Qualitative content analysis: demarcation, varieties, developments. Forum Qual Soc Res.

[45] Cabitza F, Rasoini R, Gensini GF (2017). Unintended consequences of machine learning in medicine. JAMA.

[46] Sarker IH (2022). AI-based modeling: techniques, applications and research issues towards automation, intelligent and smart systems. SN Comput Sci.

[47] Wang P (2019). On defining artificial intelligence. J Artif Gen Intelligence.

[48] Monett D, Lewis CWP, Müller VC (2018). Philosophy and Theory of Artificial Intelligence 2017.

[49] Dolata U (2008). Technologische Innovationen und sektoraler Wandel: eingriffstiefe, adaptionsfähigkeit, transformationsmuster: ein analytischer ansatz [Article in German]. Zeitschrift Soziologie.

[50] Sartori L, Theodorou A (2022). A sociotechnical perspective for the future of AI: narratives, inequalities, and human control. Ethics Inf Technol.

[51] Klein HK, Kleinman DL (2002). The social construction of technology: structural considerations. Sci Technol Hum Values.

[52] Bohr A, Memarzadeh K, Bohr A, Memarzadeh K (2020). Artificial Intelligence in Healthcare.

[53] Siala H, Wang Y (2022). SHIFTing artificial intelligence to be responsible in healthcare: a systematic review. Soc Sci Med.

[54] Tolsgaard MG, Pusic MV, Sebok-Syer SS (2023). The fundamentals of artificial intelligence in medical education research: AMEE guide no. 156. Med Teach.

[55] Sartori L, Bocca G (2022). Minding the gap(s): public perceptions of AI and socio-technical imaginaries. AI Soc.

