# Supplementary material for: Assessing AI Awareness and Identifying Essential Competencies: Insights From Key Stakeholders in Integrating AI Into Medical Education
Source: JMIR Med Educ. 2024 Jun 12;10:e58355. doi: 10.2196/58355 (PMC11238140; doi:10.2196/58355)
Supplement: Multimedia Appendix 1 [file mededu-v10-e58355-s001.docx]

| **Guiding Questions AI-Experts** | **Specific Questions** | **Follow-up Questions** |
| --- | --- | --- |
| ***General Section*** |  |  |
| 1. How old are you? 2. In which field are you working? 3. How long have you been doing this job? 4. Which gender would you describe yourself as? |  |  |
| ***General Questions (same for everyone)*** |  |  |
| 1. What comes to mind if you think of the term artificial intelligence? 2. To what extent does artificial intelligence play a role in your daily life? 3. Which experiences with AI have you already had in the healthcare sector? 4. In your opinion, what competencies should healthcare students be taught about AI? | *If so, when and where?*  *How did you perceive this?* | Could you give an example of this? Could you describe it in more detail?  Could you describe the experience in detail?  Could you elaborate on that? |
| ***Actor-specific Questions*** |  |  |
| 1. Do you perceive the extent to which AIs are integrated into healthcare as sufficient? 2. In your opinion, to what extent should there be a focus on AI in medical studies? 3. Which AI-related thematic aspects should be included (more) in health-related fields of study in the future? 4. What medical competencies would you say AIs can replace? | *At what point in the studies would you consider this focus to be useful? What forms of teaching would you consider suitable for this purpose?*  *Where do you see limitations?* |  |
| ***Outlook*** |  |  |
| 1. How do you think AI in medicine will change your professional life in the future? 2. In what areas of healthcare do you see potential to make progress using AI-related techniques in the future? 3. How useful would you consider the integration of AI-related curricula for other medical-related studies? | *What could that progress be? What challenges and limitations might arise?*  *What role could AI play in these fields (e.g. midwifery, dentistry, molecular medicine, ...)?* |  |
